# The feasibility of transpedicular screw fixation of the subaxial cervical spine in the Arab population: a computed tomography-based morphometric study

**DOI:** 10.1007/s10195-016-0396-9

**Published:** 2016-02-11

**Authors:** Osama Al-Saeed, Yousef Marwan, Osama Rabie Kombar, Ahmed Samir, Mehraj Sheikh

**Affiliations:** 1 Department of Radiology, Faculty of Medicine, Health Sciences Center, Kuwait University, PO Box 24923, Safat, 13110 Kuwait City, Kuwait; 2 Department of Radiology, Al-Amiri Hospital, Kuwait City, Kuwait; 3 Department of Orthopaedic Surgery, Al-Razi Orthopaedic Hospital, Kuwait City, Kuwait; 4 Department of Radiology, Mansoura University, Mansoura, Egypt; 5 Department of Radiology, Mubarak Al-Kabeer Hospital, Kuwait City, Kuwait

**Keywords:** Pedicle, Cervical spine, Transpedicular fixation, Screw fixation, Computed tomography, Anatomy

## Abstract

**Background:**

Transpedicular screw fixation of the cervical spine provides excellent biomechanical stability. The feasibility of inserting a 3.5-mm screw in the pedicle requires a minimum pedicle diameter of 4.5 mm. This diameter allows at least 0.5 mm bony bridge medially and laterally in order to avoid pedicle violation which can result in neurovascular complications. We aim to evaluate the feasibility of this technique in Arab people since no data are available about this population.

**Materials and methods:**

This cross-sectional study involved a retrospective review of computed tomography scans of normal cervical spines of 99 Arab adults. Ten morphometric measurements were obtained. Data were analyzed using a *p* value of ≤0.05 as the cut-off level of statistical significance.

**Results:**

Our sample included 63 (63.6 %) males and 36 (36.4 %) females, with a mean age of 35.5 ± 16.5 years. The morphometric parameters of C3–C7 spine pedicles were larger in males than in females. The outer pedicle width (OPW) was <4.5 mm in >25 % of all subjects at C3–C6 vertebrae. Statistically significant differences in the OPW between males and females were noted at C3 (*p* = 0.032) and C6 (*p* = 0.004).

**Conclusions:**

Inserting pedicle screws in the subaxial cervical spine is feasible among the majority of Arab people.

**Level of evidence:**

Level 3.

## Introduction

Numerous conditions of the cervical spine, such as trauma, deformities, tumors and osteoarthritis, require rigid fixation and solid fusion of the vertebral segments in order to achieve good treatment results. The most reliable and strongest technique for stabilization and immobilization of the spine is transpedicular screw fixation (TPSF) [1, 2]. Placing screws in the pedicles provides a better bony purchase compared to other techniques of spine fixation, leading to higher biomechanical stability [3, 4]. Nevertheless, TPSF of the cervical spine remains a difficult procedure due to the close proximity of the cervical pedicles to the vertebral artery, spinal cord and nerve roots [5, 6]. In addition, limited space is available for screw placement because of the complex anatomy of cervical spine vertebrae [7]. Therefore, the risk of complications due to screw violation of the adjacent vascular and neural structures is expected to be high when performing the operation without a clear understanding of the morphometric characteristics of the pedicles [8].

Morphometry of the cervical spine pedicles was studied before using cadavers and computed tomography (CT) scans [2, 9]. It was found that some morphometric measurements significantly differ across gender and race. This fact emphasizes the importance of studying pedicle morphometry across different populations in order to enhance the safety of TPSF surgery.

Regardless of numerous anatomical studies on cervical spine pedicles, the morphometry of this structure was never examined among Arab people. Therefore, we aim to obtain these measurements among this population in order to provide information that might help spine surgeons in fixing the cervical spines of Arab patients.

## Materials and methods

### Subjects and setting

This cross-sectional study involved a retrospective review of CT scans of the cervical spine obtained between January 2014 and December 2014 at Al-Amiri Hospital in Kuwait. Obtaining informed consent from involved patients was waived by our Research Ethics Committee. All procedures involving human participants were in accordance with the 1964 Helsinki Declaration and its later amendments. The study was approved by our local Research Ethics Committee. Inclusion criteria were patients aged at least 18 years, citizens of an Arab country, and no evidence of cervical spine congenital malformations, trauma, infection or tumor, as well as previous cervical spine surgery.

A 64-slice multidetector CT scanner (highspeed QX/i; GE Medical Systems, Milwaukee, WI, USA) with a gantry rotation speed of 0.8 s per rotation was used. Images of the cervical spine were obtained while the patients were lying supine. The coverage area of scanning included the whole cervical spine, from the base of the skull down to the upper dorsal spine, scanned in the craniocaudal direction. Slice thickness of 5 mm, pitch of 1.5, table speed of 15 mm per rotation, reconstruction interval of 2 mm, tube voltage of 120 kV, and tube current of 200 mA were used for scanning. A picture archiving and communication system workstation monitor (IMPAX, DS3000; AGFA, Mortsel, Belgium) was used to review the transferred transverse CT scans as digital images. Coronal and sagittal multiplanar images were reconstructed. The morphometric parameters of the pedicles were measured from images of multiplanar reformations.

**Fig. 1:**
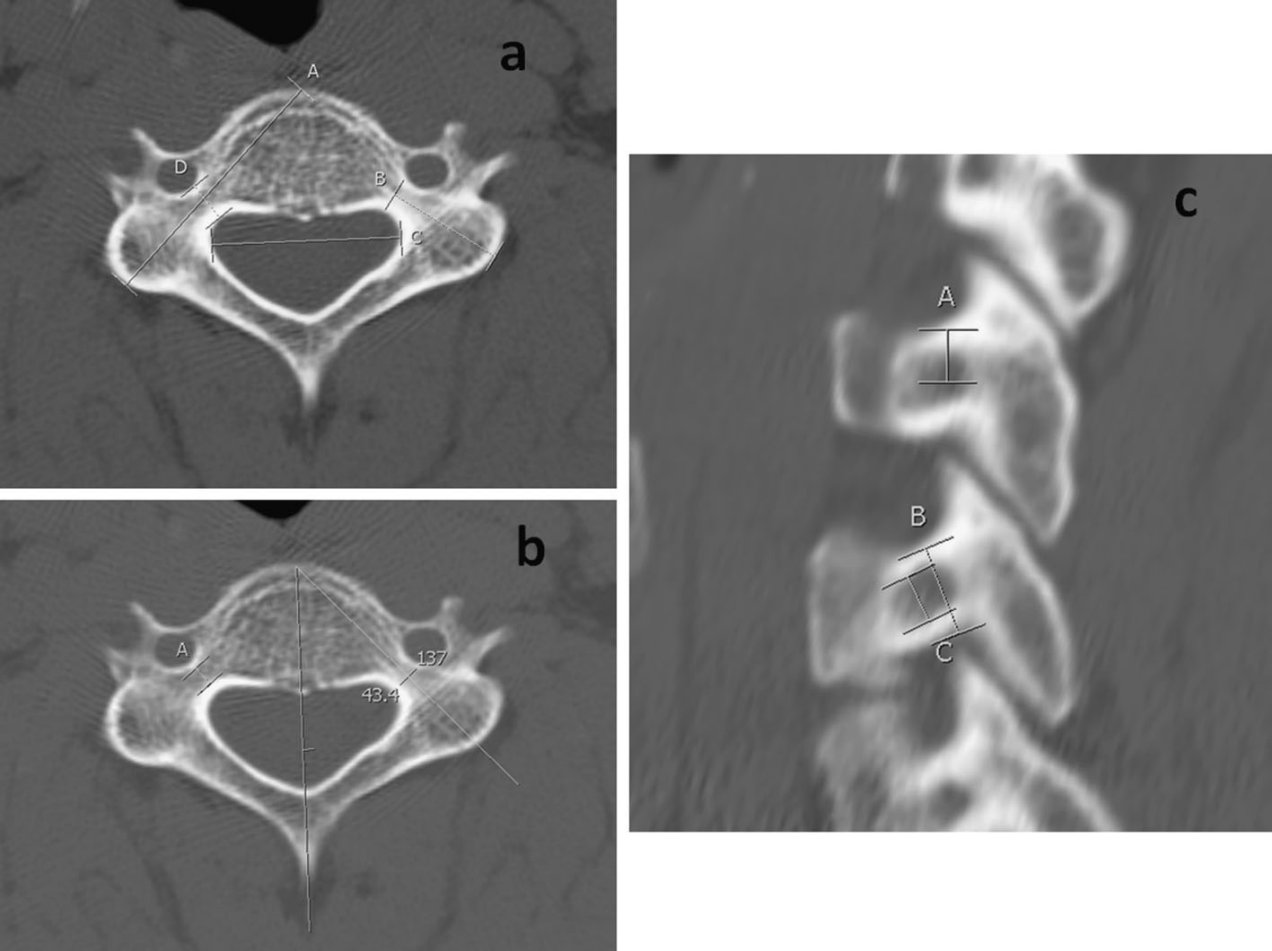
Morphometric measurements of the subaxial cervical spine pedicle. **a** Axial image of C4 vertebra on computed tomography scan showing the pedicle axis length (*A*), pedicle length (*B*), interpedicular distance (*C*) and outer pedicle width (*D*). **b** Axial image of C4 vertebra on computed tomography scan showing the inner pedicle width (*A*) and transverse angle, which in this particular case was 43.4°. **c** Sagittal reconstruction image of the cervical spine at the level of C3 and C4 showing the superior pedicle distance (*A*), outer pedicle height (*B*) and inner pedicle height (*C*)

Ninety-nine patients were eligible for inclusion. Nine morphometric measurements were obtained for each pedicle, starting from C3 to C7, including both the right and left side (Table 1; Fig. 1). In addition to these measurements, the interpedicular distance (IPD) was also measured. The pedicle sagittal angle was not measured in our current study because of the variation in the technique of measuring this angle among previous investigators making it an unreliable measure [9–12].

**Table 1:** Morphometric parameters of cervical spine pedicles

Parameter	Definition
Outer pedicle height	Outer superior to inferior diameter of the pedicle isthmus
Outer pedicle width	Outer medial to lateral diameter of the pedicle isthmus
Inner pedicle height	Superior to inferior diameter of the cancellous core of the pedicle isthmus
Inner pedicle width	Medial to lateral diameter of the cancellous core of the pedicle isthmus
Pedicle axis length	Distance from the posterior projective points of the pedicle axis on the lateral mass to the anterior margin of the vertebral body
Pedicle length	Distance from the posterior projective points of the pedicle axis on the lateral mass to the junction of the vertebral body and pedicle
Superior pedicle distance	Distance from the inferior edge of the superior facet to the posterior projective points of the pedicle axis on the lateral mass
Lateral pedicle distance	Distance from the lateral edge of the lateral mass to the posterior projective points of the pedicle axis on the lateral mass
Pedicle transverse angle	The angle between the pedicle axis projection and the anatomic sagittal plane
Interpedicular distance	Distance between the most medial point of the pedicle isthmus in the transverse plane

A total of 990 pedicles (198 pedicles at each vertebral level) were evaluated in this study. In order to assess the intra-observer repeatability and inter-rater reproducibility for these parameters, the measurements were repeated in 20 patients at 1 week after the initial assessment by the same radiologist as well as an independent investigator.

### Statistical analysis

The Statistical Package for Social Sciences version 17.0 (SPSS Inc, Chicago, IL, USA) was used for data analysis. Descriptive results, including frequencies, percentages, means and standard deviations, were measured for all variables. Student *t* test was used to assess the association between patient gender and pedicle morphometric parameters. This test was used because gender is a binary qualitative variable, and the morphometric variables are normally distributed quantitative variables. The outer pedicle width (OPW) was re-coded into a binary qualitative variable, and the chi-squared test was used to assess the association between OPW and patient gender, since this measure is considered to be the most important when planning TPSF surgery. For statistical significance, a *p* value of ≤0.05 was used as the cut-off level. Moreover, inter- and intraclass correlation coefficients were calculated in order to assess the reliability of the morphometric measurements that were obtained by our two radiologists in this study [13].

## Results

**Fig. 2:**
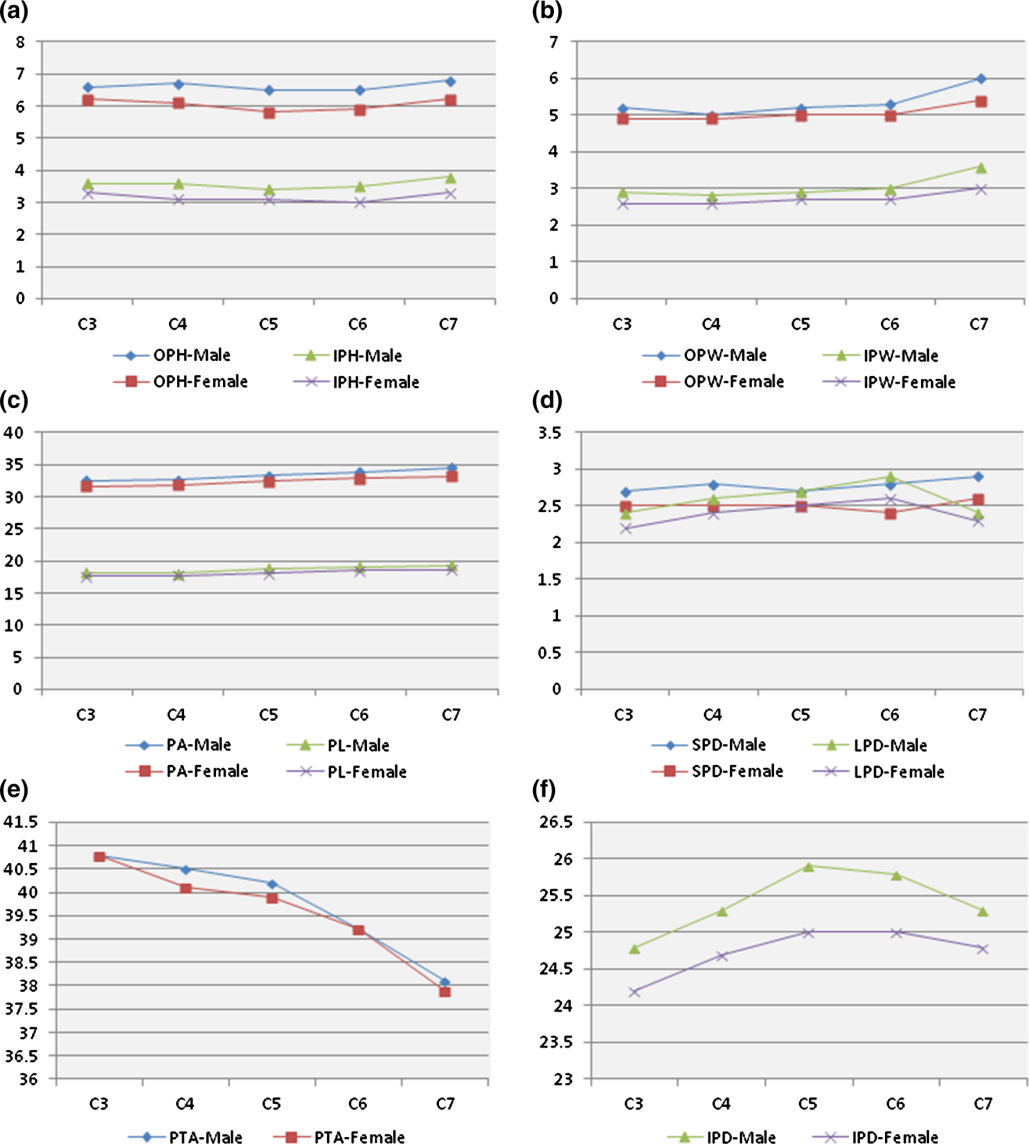
Morphometric findings of the subaxial cervical spine among Arab adults (*N* = 99; 990 pedicles). **a** Outer pedicle height (OPH) and inner pedicle height (IPH), **b** outer pedicle width (OPW) and inner pedicle width (IPW), **c** pedicle axis length (PA) and pedicle length (PL), **d** superior pedicle distance (SPD) and lateral pedicle distance (LPD), **e** pedicle transverse angle (PTA), **f** interpedicular distance (IPD)

Our sample included 63 (63.6 %) males and 36 (36.4 %) females, with a mean age of 35.5 ± 16.5 years. The mean age for males was 33.1 ± 14.4 years, while the mean age for females was 39.7 ± 19.1 years (*p* = 0.012). All morphometric findings are shown in Tables 2, 3 and Fig. 2.

**Table 2:** Morphometric findings of the subaxial cervical spine of Arab adults (*N* = 99; 990 pedicles)

Parameter	All patients		Males		Females		*p* value
	Mean ± SD	(Range)	Mean ± SD	(Range)	Mean ± SD	(Range)	
C3							
OPH (mm)	6.4 ± 0.9	(3.6–9.1)	6.6 ± 0.8	(3.8–9.1)	6.2 ± 0.9	(3.6–7.5)	0.003
OPW (mm)	5.1 ± 1.3	(1.8–7.7)	5.2 ± 1.2	(2.4–7.5)	4.9 ± 1.4	(1.8–7.7)	0.057
IPH (mm)	3.5 ± 1.0	(1.5–8.0)	3.6 ± 1.0	(1.9–8.0)	3.3 ± 1.0	(1.5–5.9)	0.037
IPW (mm)	2.8 ± 1.1	(0.6–7.2)	2.9 ± 1.0	(0.7–6.0)	2.6 ± 1.1	(0.6–7.2)	0.100
PA (mm)	32.2 ± 2.4	(23.2–39.7)	32.5 ± 2.5	(26.6–39.7)	31.6 ± 2.2	(23.2–37.2)	0.017
PL (mm)	18.0 ± 3.3	(11.2–36.8)	18.2 ± 3.5	(11.6–36.8)	17.6 ± 3.0	(11.2–28.6)	0.181
SPD (mm)	2.6 ± 0.6	(1.2–4.4)	2.7 ± 0.6	(1.2–4.4)	2.5 ± 0.5	(1.3–3.7)	0.027
LPD (mm)	2.3 ± 0.8	(0.8–5.3)	2.4 ± 0.8	(0.8–4.6)	2.2 ± 0.9	(0.9–5.3)	0.221
PTA (°)	40.8 ± 3.4	(27.4–48.3)	40.8 ± 3.2	(32.7–48.3)	40.8 ± 3.8	(27.4–47.1)	0.910
IPD (°)	24.6 ± 1.8	(12.7–32.1)	24.8 ± 1.6	(20.9–32.1)	24.2 ± 2.1	(12.7–28.3)	0.021
C4							
OPH (mm)	6.5 ± 1.0	(3.6–9.2)	6.7 ± 0.9	(3.7–9.2)	6.1 ± 1.0	(3.6–7.8)	<0.001
OPW (mm)	5.0 ± 1.3	(2.1–7.9)	5.0 ± 1.2	(2.3–7.9)	4.9 ± 1.4	(2.1–7.3)	0.334
IPH (mm)	3.4 ± 1.0	(1.6–7.9)	3.6 ± 1.0	(1.8–7.9)	3.1 ± 0.9	(1.6–5.2)	0.004
IPW (mm)	2.7 ± 1.0	(0.6–6.3)	2.8 ± 1.0	(0.7–6.1)	2.6 ± 1.0	(0.6–6.3)	0.118
PA (mm)	32.3 ± 2.7	(22.8–41.0)	32.6 ± 2.6	(26.9–41.0)	31.8 ± 2.7	(22.8–38.2)	0.035
PL (mm)	18.0 ± 3.6	(12.2–35.0)	18.1 ± 3.6	(12.2–34.9)	17.7 ± 3.7	(12.5–35.0)	0.462
SPD (mm)	2.7 ± 0.6	(1.2–4.4)	2.8 ± 0.7	(1.3–4.4)	2.5 ± 0.5	(1.2–3.9)	0.004
LPD (mm)	2.5 ± 1.0	(1.2–6.7)	2.6 ± 1.0	(1.2–6.7)	2.4 ± 1.0	(1.2–5.2)	0.278
PTA (°)	40.4 ± 3.8	(19.5–48.5)	40.5 ± 4.1	(19.5–48.5)	40.1 ± 3.4	(29.5–46.0)	0.515
IPD (mm)	25.1 ± 1.7	(11.7–30.3)	25.3 ± 1.5	(21.2–30.3)	24.7 ± 2.0	(11.7–28.7)	0.025
C5							
OPH (mm)	6.2 ± 1.0	(2.8–9.0)	6.5 ± 0.9	(2.8–9.0)	5.8 ± 1.0	(4.0–7.9)	<0.001
OPW (mm)	5.1 ± 1.2	(2.5–7.3)	5.2 ± 1.2	(3.1–7.3)	5.0 ± 1.2	(2.5–7.3)	0.191
IPH (mm)	3.3 ± 1.0	(1.2–7.5)	3.4 ± 1.0	(1.2–7.5)	3.1 ± 0.9	(1.4–5.1)	0.042
IPW (mm)	2.8 ± 0.9	(0.9–5.9)	2.9 ± 1.0	(1.1–5.9)	2.7 ± 0.9	(0.9–5.2)	0.080
PA (mm)	33.0 ± 2.9	(21.2–41.1)	33.3 ± 2.9	(22.8–41.1)	32.4 ± 3.0	(21.2–38.3)	0.045
PL (mm)	18.6 ± 3.6	(12.5–38.8)	18.8 ± 3.6	(12.5–38.8)	18.1 ± 3.6	(12.9–33.0)	0.201
SPD (mm)	2.6 ± 0.6	(1.2–5.1)	2.7 ± 0.7	(1.2–5.1)	2.5 ± 0.5	(1.2–3.3)	0.005
LPD (mm)	2.6 ± 1.2	(0.8–8.5)	2.7 ± 1.2	(1.2–8.5)	2.5 ± 1.1	(0.8–5.9)	0.152
PTA (°)	40.1 ± 4.2	(17.5–51.8)	40.2 ± 4.3	(17.5–51.8)	39.9 ± 4.1	(26.3–48.0)	0.590
IPD (mm)	25.6 ± 1.9	(11.2–30.8)	25.9 ± 1.6	(21.5–30.8)	25.0 ± 2.2	(11.2–29.0)	0.004
C6							
OPH (mm)	6.3 ± 1.0	(3.1–8.4)	6.5 ± 0.9	(3.1–8.4)	5.9 ± 0.9	(3.6–7.3)	<0.001
OPW (mm)	5.2 ± 1.1	(2.9–7.7)	5.3 ± 1.1	(2.9–7.7)	5.0 ± 1.0	(3.1–7.2)	0.088
IPH (mm)	3.3 ± 0.9	(1.5–6.8)	3.5 ± 0.9	(1.6–6.8)	3.0 ± 0.9	(1.5–5.4)	<0.001
IPW (mm)	2.9 ± 0.9	(1.0–6.1)	3.0 ± 0.9	(1.0–6.1)	2.7 ± 0.8	(1.1–4.9)	0.035
PA (mm)	33.5 ± 2.9	(22.9–40.2)	33.9 ± 2.9	(25.7–40.2)	32.8 ± 2.9	(22.9–38.2)	0.014
PL (mm)	18.9 ± 3.6	(12.9–38.1)	19.1 ± 3.7	(12.9–38.1)	18.5 ± 3.4	(13.3–33.0)	0.286
SPD (mm)	2.6 ± 0.7	(1.2–4.6)	2.8 ± 0.7	(1.2–4.6)	2.4 ± 0.6	(1.2–4.0)	0.001
LPD (mm)	2.8 ± 1.2	(1.2–8.4)	2.9 ± 1.2	(1.2–8.4)	2.6 ± 1.1	(1.2–5.2)	0.145
PTA (°)	39.2 ± 3.7	(22.5–47.5)	39.2 ± 3.8	(22.5–46.7)	39.2 ± 3.5	(27.4–47.5)	0.868
IPD (mm)	25.5 ± 1.9	(11.7–31.1)	25.8 ± 1.7	(21.3–31.1)	25.0 ± 2.2	(11.7–28.4)	0.007
C7							
OPH (mm)	6.6 ± 0.9	(3.2–9.0)	6.8 ± 0.8	(4.5–8.9)	6.2 ± 0.9	(3.2–9.0)	<0.001
OPW (mm)	5.8 ± 1.2	(3.1–9.0)	6.0 ± 1.2	(3.1–9.0)	5.4 ± 1.0	(3.7–7.6)	0.001
IPH (mm)	3.6 ± 0.9	(1.6–7.1)	3.8 ± 0.8	(2.1–7.1)	3.3 ± 0.9	(1.6–6.4)	<0.001
IPW (mm)	3.4 ± 0.9	(1.4–6.2)	3.6 ± 0.9	(2.1–6.2)	3.0 ± 0.7	(1.4–4.8)	<0.001
PA (mm)	34.0 ± 3.3	(23.5–43.3)	34.5 ± 3.4	(23.5–43.3)	33.2 ± 3.0	(23.7–39.4)	0.012
PL (mm)	19.1 ± 3.6	(12.3–41.1)	19.3 ± 3.7	(12.3–41.1)	18.6 ± 3.2	(13.3–32.0)	0.155
SPD (mm)	2.8 ± 0.7	(1.2–4.7)	2.9 ± 0.8	(1.2–4.7)	2.6 ± 0.5	(1.7–3.9)	0.006
LPD (mm)	2.4 ± 0.9	(0.9–5.7)	2.4 ± 0.9	(0.9–5.4)	2.3 ± 0.7	(0.9–4.6)	0.414
PTA (°)	38.1 ± 3.5	(26.4–48.0)	38.1 ± 3.4	(27.5–46.2)	37.9 ± 3.5	(26.4–48.0)	0.679
IPD (mm)	25.1 ± 1.9	(11.9–30.7)	25.3 ± 1.6	(21.7–30.7)	24.8 ± 2.4	(11.9–29.1)	0.083

*SD* standard deviation, *OPH* outer pedicle height, *OPW* outer pedicle width, *IPH* inner pedicle height, *IPW* inner pedicle width, *PA* pedicle axis length, *PL* pedicle length, *SPD* superior pedicle distance, *LPD* lateral pedicle distance, *PTA* pedicle transverse angle, *IPD* interpedicular distance

Student *t* test was used to calculate the *p* value

**Table 3:** Outer pedicle width of the subaxial cervical spine pedicles of Arab adults (*N* = 99; 990 pedicles)

Outer pedicle width	All patients		Males		Females		*p* value
	*N*	%	*N*	%	*N*	%	
C3							0.032
<4.5 mm	69	34.8	37	29.4	32	44.4	
≥4.5 mm	129	65.2	89	70.6	40	55.6	
C4							0.323
<4.5 mm	79	39.9	47	37.3	32	44.4	
≥4.5 mm	119	60.1	79	62.7	40	55.6	
C5							0.256
<4.5 mm	67	33.8	39	31.0	28	38.9	
≥4.5 mm	131	66.2	87	69.0	44	61.1	
C6							0.004
<4.5 mm	51	25.8	24	19.0	27	37.5	
≥4.5 mm	147	74.2	102	81.0	45	62.5	
C7							0.149
<4.5 mm	29	14.6	15	11.9	14	19.4	
≥4.5 mm	169	85.4	111	88.1	58	80.6	

Chi-squared test was used to calculate the *p* value

### Pedicle height and width (Tables 2, 3; Fig. 2a, b)

The mean outer pedicle height (OPH), OPW, inner pedicle height (IPH) and inner pedicle width (IDW) were larger among males at all levels. The most significant differences were observed at C7. A statistically significant difference in OPH and IPH between males and females was noted from C3 to C7 (*p* values ranged from 0.042 to <0.001). The OPW was significantly larger at C7 only (*p* = 0.001), while the IPW was significantly larger at C6 (*p* = 0.035) and C7 (*p* < 0.001).

A larger percentage of males compared to females had an OPW of ≥4.5 mm at C3–C7 levels (Table 3), which was statistically significant at C3 (*p* = 0.032) and C6 (*p* = 0.004). At C4, 79 (39.9 %) of the subjects had an OPW of <4.5 mm. On the other hand, only 29 (14.6 %) subjects had an OPW of <4.5 mm at C7.

### Pedicle axis length and pedicle length (Table 2; Fig. 2c)

The mean pedicle axis (PA) length was significantly larger among males at all vertebral levels assessed in this study (*p* value ranged from 0.045−0.012). The pedicle length (PL) was also larger among males at all levels; however, this difference was not statistically significant. Among all subjects, the smallest PL (11.2 mm) was at C3, while the largest (41.1 mm) was at C7. The overall mean of both PA and PL consistently increased from the cephalad (C3) to the caudad (C7).

### Superior and lateral pedicle distances (Table 2; Fig. 2d)

A statistically significant difference was noted in the superior pedicle distance (SPD) between males and females at all C3–C7 levels (*p* value ranged 0.027–0.001); males also had larger SPD than females. The lateral pedicle distance (LPD) was larger among males; however, this difference was not statistically significant. From C3 to C7, the smallest SPD was 1.2 mm. On the other hand, the smallest LPD (0.8 mm) was seen at both C3 and C5. C5 had the largest SPD (5.1 mm) and LPD (8.5 mm).

### Pedicle transverse angle (Table 2; Fig. 2e)

There was no statistically significant difference between males and females in the pedicle transverse angle (PTA). The mean PTA of males and females was equal at C3, and larger among males at all other levels. The largest (51.8°) and smallest (17.5°) PTA was seen at C5. The overall mean of PTA consistently decreased from the cephalad (C3) to the caudad (C7).

### Interpedicular distance (Table 2; Fig. 2f)

The mean IPD was larger among males at all vertebral levels. This was statistically significant for all levels except C7 (*p* values ranged from 0.083−0.004). The smallest IPD was seen in C5, while the largest was at C3.

### Reliability

The inter- and intraclass correlation coefficients were between 0.74 and 0.99 for all morphometric parameters. This indicates that reproducibility and repeatability were substantial to almost perfect, respectively.

## Discussion

TPSF of the cervical spine was proposed because of the limited biomechanical stability of the commonly used posterior plating techniques. The preferred site of screw placement for posterior plating is the lateral mass [14]. The small amount of bony purchase available in the lateral mass results in biomechanical instability leading to loosening or avulsion of the screw [3]. A significantly higher resistance to pull-out forces, lower rate of loosening and higher strength after fatigue testing were observed with cervical pedicle screws compared to lateral mass screws during biomechanical investigations [3, 15]. In addition, screw-related complications, such as screw loosening, loss of reduction, pseudarthrosis and revision surgery were more commonly reported with lateral mass screws in the subaxial spine [16]. Accordingly, TPSF is being commonly used nowadays.

Although TPSF provides excellent biomechanical stability to the cervical spine, violation of the pedicle cortex is possible. Screw perforation was seen in up to 29.8 % of cases; however, this rarely caused significant complications [16–18]. As a consequence of screw perforation, vertebral artery and nerve root injuries are possible. These complications were reported in a limited number of cases in the literature [16–18]. In order to avoid these serious complications, surgeons should have a proper understanding of the anatomy of the cervical spine and perform appropriate preoperative planning using CT scans for each individual patient. Moreover, the use of intraoperative CT scans, 3-dimensional fluoroscopy and other forms of navigation systems have been shown to reduce the rate of screw perforation and complications [19, 20].

This is the first study to provide CT scan-based morphometric evaluation of the subaxial cervical spine pedicles of Arab adults. Our results revealed that the morphometric parameters of the C3–C7 cervical spine pedicles were larger in males than in females. This finding is similar to results found among other ethnic groups [2, 9, 21, 22]. Spine surgeons should carefully take into account such gender differences before performing TPSF surgery. Moreover, the CT scan dimensions of the subaxial pedicles of our subjects were found to be slightly different when compared to Asians, Europeans and Americans [2, 9]. Height and width were noted to be smaller in Arab people compared to Asians and European/Americans, highlighting the importance of thorough preoperative planning for Arab patients undergoing TPSF of the cervical spine.

The feasibility of inserting a 3.5-mm screw in the pedicle requires a minimum pedicle diameter of 4.5 mm. This diameter allows at least 0.5 mm bony bridge medially and laterally in order to avoid pedicle violation which can result in neurovascular complications [1, 12, 16, 22, 23]. Based on data from previous reports and our current study, the OPW is considered to be the most important parameter in assessing the feasibility of the TPSF technique [1, 2, 9, 12, 24, 25]. This is because the OPH is larger than the width. An OPW of <4.5 mm was seen in more than one-third of males at C4, and more than one-third of females at C3–C6 among our subjects. This indicates that TPSF of the cervical spine is more feasible among Arab males compared to Arab females. Moreover, this technique of fixation appeared to be more applicable at lower cervical vertebral levels in all ethnic groups [2, 9]. The mean OPW of the subaxial spine of Asians ranged from 5.26−6.63 mm, while that of Europeans/Americans ranged from 5.17−6.64 mm [9]. In our current study, this morphometric measurement ranged from 5.0−5.8 mm among Arab people. These results indicate that a preoperative CT scan evaluation is mandatory for Arab patients before TPSF surgery, especially if the patient is female and the fracture involves higher levels of the cervical spine. Another morphometric finding which can help spine surgeons is the PTA, which had a mean of approximately 40° at all C3–C7 levels. This indicates that the angulation of screw placement at the transverse plane should be directed medially in Arab patients to avoid complications. Other studies reported very close values, in which the PTA of the subaxial spine ranged from 37.1° to 49° and 38.7° to 48.8° among Asians and Europeans/Americans, respectively [2, 9]; this means that cervical spine pedicle screw insertion should always be directed medially regardless of the patient’s ethnicity [2, 9, 21, 22].

Although this study provides important information about the morphometry of the subaxial cervical spine pedicles, it has a limitation. Possible differences in the morphometric parameters might exist between Arab people from different geographic regions (i.e., South West Asia vs North Africa). Our study included Arab people from both regions; however, we did not document this feature during our data collection.

In conclusion, the morphometry of the pedicles of the subaxial cervical spine of Arab people shared some similarities and differences compared to other ethnic groups. For the majority of our subjects, inserting screws in the pedicles of C3–C7 vertebrae is feasible. In order to avoid serious intraoperative complications, spine surgeons should carefully assess the morphometry of the pedicles preoperatively for Arab patients undergoing TPSF surgery at the level of the cervical spine.
